# Biomass, Essential Oil Yield, and Composition of Marjoram as Influenced by Interactions of Different Agronomic Practices under Controlled Conditions

**DOI:** 10.3390/plants12010173

**Published:** 2022-12-30

**Authors:** Mantwampe Johleen Malaka, Nadia Alcina Araya, Puffy Soundy, Christian Phillipus du Plooy, Hintsa Tesfamicael Araya, Willem Sternburg Jansen Van Rensburg, Eric Watkinson, Ellis Levember, Ebrahim Wadiwala, Stephen Oluwaseun Amoo

**Affiliations:** 1 Agricultural Research Council-Vegetable, Industrial and Medicinal Plants, Private Bag X293, Pretoria 0001, South Africa; 2 Department of Crop Sciences, Tshwane University of Technology, Private Bag X680, Pretoria 0001, South Africa; 3 Department of Science and Innovation, Private Bag X894, Pretoria 0001, South Africa; 4 South African Essential Oils Business Incubator, 19 Mountain Street, Derdepoort 0186, Pretoria, South Africa; 5 Council for Scientific and Industrial Research, P.O. Box 395, Meiring Naude Road, Brummeria, Pretoria 0001, South Africa; 6 Department of Botany and Plant Biotechnology, University of Johannesburg, P.O. Box 524, Auckland Park, Johannesburg 2006, South Africa; 7 Indigenous Knowledge Systems Centre, Faculty of Natural and Agricultural Sciences, North-West University, Private Bag X2046, Mmabatho 2790, South Africa

**Keywords:** Lamiaceae, nitrogen fertilization, irrigation, soil type, air temperature, herbage yield, essential oil, *Origanum marjorana*

## Abstract

*Origanum marjorana* L. has been valued for centuries for its flavoring attributes and therapeutic properties. The growing demand for its various applications necessitates optimizing agronomic practices for its production. A glasshouse pot trial was conducted to identify optimum agronomic practices for increased herbage and oil yield, as well as oil quality. The effects of varying air temperature regimes (low, medium, and high levels), irrigation (low, medium, and high levels), nitrogen fertilizer application (N = 100, 150, and 200 kg/ha), and soil type (sandy loam, sandy clay loam, and loamy sand) on the productivity of marjoram plants were investigated. The results showed an increase in plant growth and herbage yield as well as chlorophyll content under conditions of high air temperature, low irrigation, and moderate to high nitrogen level applied to sandy loam soil, with an increase in oil yield with loamy sand soil. The major compounds observed in marjoram essential oil were terpinene-4-ol (22.63–36.72%) and (Z)-β-terpineol (6.85–16.60%), in which terpinene-4-ol was not found to be within the International Organization for Standardization (ISO) limits of acceptability while (Z)-β- terpineol had no reference limits available. A promising performance of marjoram cultivation under high regimes of air temperature (16.7 to 36.6 °C), nitrogen fertilization (200 kg ha^−1^ N), and low irrigation (up to 60% soil water depletion from field capacity) on sandy loam soils was demonstrated for improved crop productivity.

## 1. Introduction

In modern agriculture and trade, the list of economically important plants has been expanded beyond feed, traditional foods, and fibre crops to progressively include species containing chemical compounds that have desired aromatic or medicinal properties, or serve as sources of material for the perfume, food, and chemical industries [1,2,3]. One of these plants is marjoram (*Origanum majorana* L.), which is a member of the Lamiaceae family. It is widely cultivated in Mediterranean countries. Marjoram is a perennial bushy plant with oblique rhizome, hairy shrub-like stalks, oppositely arranged dark green oval leaves, and white or red flowers in clustered bracts [4]. Marjoram leaves possess an attractive fragrance. The fresh or dried highly aromatic leaves and flowering tops of marjoram are widely used as a spice to flavour, improve sensory characteristics, and extend the shelf life of many foods [5]. In addition, as a valuable medicinal and aromatic plant, it has strong antimicrobial and antioxidant properties against several bacterial infections and mycotoxigenic fungi [4,6,7]. Its use in the prevention and treatment of glaucoma due to its antioxidant content has also been advocated [8]. Some authors have reported about the effectiveness of marjoram extracts in reducing lipid oxidation [9], colour loss, and microbial growth [10,11] within certain types of meats. It is considered as an important tradable plant/plant product because of its great potential for industrial and pharmaceutical applications.

The medicinal properties of plants are due to phytochemical components, including essential oils, produced during secondary metabolism [12]. Essential oils are concentrated hydrophobic liquids characterized by a strong aroma containing volatile chemical compounds from plants [13,14]. They are synthesized by different plant organs (such as flowers, buds, seeds, leaves, twigs, bark, fruits, and roots) and are stored in secretory cells, cavities, canals, epidermal cells, or glandular trichomes [15]). They are gaining popularity as a natural, safe, and cost-effective therapy for a number of health concerns due to their antidepressant, stimulatory, detoxifying, antibacterial, antiviral, and calming properties, amongst others [16]. The oil is isolated by steam distillation from different plant parts. Marjoram herb produces an essential oil that is yellowish in colour and yields about 0.5 to 3% [17]. In general, carvacrol, thymol, terpinen-4-ol [18,19,20], trans-caryophyllene, gamma-terpinene, and *p*-cymene [21,22] represent major contributing compounds of this essential oil. The growing demand in industrialized countries for natural products, as alternatives to synthetic compounds, has created a niche market for medicinal and aromatic plants.

The entry into the essential oil market depends not only on demand, but also on achieving a competitive price for quality production at the required scale or volume of production. These requirements necessitate having optimum conditions in place for the cultivation of these crops. The various factors that influence crop growth, essential oil yields, and the final composition of essential oils in the plants include cultivation practices [23,24,25,26], environmental conditions [27,28,29], and genetic factors [30,31,32]. The influence of water stress and nitrogen fertilizer on plant growth, herbage and oil yield, and the quality of different *Origanum* species has been assessed previously [33,34,35,36,37]. Water stress led to a decrease in plant growth due to negative influences on the photosynthesis and transpiration processes, as well as a change in essential oil yield and composition [33,34,35,36,37]. An increase in irrigation intervals had a negative effect on *O. vulgare* morphological traits, but did not significantly affect the essential oil content and yield [38]. Changes due to different soil water levels in biomass production and essential oil profile of four Lamiaceae species including marjoram have been reported [39]. Increased nitrogen fertilizer was found relevant for improved essential oil crop production in terms of vegetative growth and the yield of fresh and dry leaves, as well as volatile oil [40,41,42]. In another study evaluating the influence of four nitrogen application levels at 0, 40, 80, and 120 kg ha^−1^, nitrogen application significantly affected the herbage yield of *Origanum vulgare* with 80 kg/ha N resulting in optimal plant growth [23]. Nitrogen fertilizer application also significantly increased the essential oil yield of *O. syriacum* [43].

Air temperature can influence plant growth, herbage yield, essential oil composition, and the yield of essential oil crops [44,45,46,47]. Increased air temperatures from 18 °C to 27 °C resulted in increased plant height and herbage yield in *Ocimum basilicum*, *Salvia officinalis*, *Rosmarinus officinalis*, and *Eruca vesicaria* [48]. The aspect of soil type on growth, productivity, and essential oil composition for many medicinal and aromatic plants is less studied. Nonetheless, soil type influenced the biomass yield, essential oil yield, and essential oil chemical composition of *O. syriacum* [49]. Similarly, Aziz et al. [50] observed that soil type affected *Thymus vulgaris* plant growth, essential oil yield, and its main constituents, with calcareous soil reported as the best for enhancing growth and yield, followed by clay loam and sandy loam. Soil type also influenced the essential oil composition of cultivated *Rosmarinus officinalis* [51].

Despite the diverse potential uses of marjoram, very little has been done to evaluate its performance under a range of agronomic practices in relation to plant growth, physiology, essential oil yield, and chemical composition. This indicates that the current knowledge about its agronomy is neither complete nor conclusive. The paucity of information limits further exploitation of this economically important spice, aromatic, and medicinal herb. To help in filling the aforementioned research and development gaps, this experiment was carried out with the objective of evaluating the response of marjoram to the interrelation of factors including different types of soil, air temperature, irrigation regimes, and nitrogen fertilization application rates, on its growth parameters, herbage yield, and essential oil composition.

## 2. Results and Discussion

### 2.1. Growth and Physiological Parameters

The analysis of variance (ANOVA) revealed variable significances within the main effects of air temperature and irrigation regimes, nitrogen application rate, and soil texture, as well as their interactions. Therefore, a hypothesis test was conducted to compare the mean square and *p*-values of plant height, number of branches, leaf chlorophyll content, leaf temperature, herbage yield, and leaf area under all tested factors and their interactions (Table 1). Based on the results presented in Table 1, leaf chlorophyll content, herbage yield and leaf area were the only traits significantly affected by an interaction of all the agronomic factors investigated.

#### 2.1.1. Plant Height

Considering the interaction between the main factors (Table 1), the results showed the significant interaction effects of several combinations of factors on plant height. The three combinations that have significant interaction effects are (i) air temperature, soil type, and nitrogen fertilizer application, (ii) irrigation regime, air temperature, and nitrogen fertilizer application, and (iii) irrigation regime, air temperature, and soil type (Table 1 and Table 2). The highest average plant heights, 47.17 cm and 46.79 cm, were obtained with plants grown under high air temperatures with a fertilizer application rate of 100 kg/ha N to sandy loam soil, and plants grown under the medium air temperature regime with a 100 kg/ha N application rate and a high level of irrigation, respectively (Table 2). The lowest average plant height (29.61 cm) was found with plants grown under medium air temperature and irrigation combined with a fertilizer application rate of 100 kg/ha N (Table 2). When considering the significant interaction effect of irrigation regimes, air temperature, and soil type on plant height, medium and high irrigation regimes in interactions with low and medium temperature levels on sandy clay loam soil, and high irrigation regime in interactions with medium and high temperatures on sandy loam soil resulted in the highest average plant height (41.44–46.61 cm). In this study, increased plant height was supported by high temperatures combined with sandy loam soil. Soil type and air temperature influence the physiological processes such as photosynthesis, respiration, and subsequent crop yield. For example, subjecting *Ocimum basilicum* to high-temperature stress resulted in an increase in metabolic processes, gas exchanges, and morphological changes such as plant height [52]. Similarly, a positive impact of increased growth temperatures culminating in increased net photosynthesis rate, transpiration rate, and stomatal conductance, with resultant benefits on yield was reported in *O*. *basilicum* [53].

Another study on oregano, sage, lemon balm, and rosemary also reported increased plant height when increasing the temperature from 18 °C to 27 °C [48]. An increased irrigation interval from weekly to every two weeks significantly reduced *O. vulgare* plant height possibly due to decreased cell enlargement or a reduction in stomatal conductance [38]. In a study on the effect of soil type and seasonal variation on the growth of *Artemisia annua* L., it was indicated that clay loamy soil significantly increased vegetative growth characters [54].

#### 2.1.2. Number of Branches per Plant

A significant interaction effect was observed between soil type, air temperature and irrigation regimes on the number of branches per plant (Table 1). As indicated in Table 3, the highest number of branches (9.77) was produced on sandy loam soils with high levels of irrigation and air temperature, although this treatment did not produce a significantly higher number of branches than those produced with high levels of irrigation and air temperature on sandy clay loam (8.77) or loamy sand soils (9.20). On the contrary, medium levels of irrigation and air temperature with sandy clay loam soil produced the lowest number of branches (4.34). In this study, it is noteworthy that the number of branches increased under a high level of irrigation, regardless of the soil type and air temperature regime, while the plants grown under a low level of irrigation had fewer branches. The higher number of branches per plant under the high level of irrigation might be due to sufficient water availability, resulting in an increase in water content, total water potential, and cell enlargement, as well as plant growth [50]. In this context, a high level of irrigation enabled marjoram plants to maintain their normal physiological processes. An earlier study on the effect of irrigation intervals on the growth parameters of *Origanum vulgare* reported a decrease in plant growth when increasing irrigation intervals, and this could be due to reduced cell growth or a decrease in photosynthesis, which could interfere with the structure of the canopy during longer irrigation intervals [38].

#### 2.1.3. Chlorophyll Content

There was a significant interaction effect of air temperature, irrigation regimes, soil type, and nitrogen fertilization application on chlorophyll content (Table 1). The highest leaf chlorophyll content (35.27) was recorded in plants grown under low irrigation and high temperature levels with a fertilizer application rate of 150 kg/ha N to sandy clay loam soil, while there was a significant decrease to 23.93 under medium levels of irrigation and air temperature with a nitrogen application rate of 150 kg/ha N to sandy loam soil (Table 4). Subjecting marjoram plants to low irrigation and air temperature regimes generally resulted in reduced chlorophyll content. This reduction in chlorophyll content may be due to decreased metabolic functions that induce growth and development, and the production of metabolites to protect the plants. The total chlorophyll content of *Capsicum frutescens* was also significantly decreased by water stress [55]. Climate, soil, and phylogeny exert only a small effect on the spatial variation of chlorophyll in natural forces [56].

#### 2.1.4. Leaf Temperature

There was no significant interaction effect of any of the factors (irrigation and air temperature regimes, nitrogen application rate, and soil type) on leaf temperature (Table 1). The major factors significantly influencing this variable were nitrogen fertilizer application rate and irrigation regimes as individual factors (Table 1 and Table 5). The upper values of leaf temperature were exhibited under a 100 to 150 kg/ha N fertilizer application rate and a low to medium irrigation regime (Table 5). Thus, a relatively low nitrogen fertilizer application rate resulted in a higher leaf temperature, which could be attributed to the development of a lower number of branches under such conditions, thus promoting higher radiation interception throughout the canopy. Similarly, N-deficient spring wheat plants generally had higher leaf temperatures, which enhanced crop maturity [57]. On the other hand, higher N fertilizer applications significantly lowered leaf temperatures of *Oryza sativa* [58]. While considering the effect of irrigation regimes on leaf temperature, there appeared to be an inverse relationship (Table 5). Leaf temperature decreased with an increase in irrigation regime. When plants transpire, water evaporates from the leaf cell and atmosphere interface, and this exothermic process releases energy into the atmosphere, thereby cooling the plant leaves [59].

#### 2.1.5. Herbage Yield

A highly significant interactive effect between air temperature and irrigation regimes, nitrogen fertilizer application, and soil type was recorded on herbage yield (Table 1). Low irrigation and high air temperature levels linked with a nitrogen application rate of 200 kg/ha N to sandy loam soils recorded the highest herbage yield of 157.3 g/plant (Table 6). However, this was not significantly higher than that of medium irrigation, low temperature, and a 100 kg/ha N application to sandy clay loam soils (148.3 g/plant) or loamy sand (146.3 g/plant). The lowest recorded herbage yield of 53.3 g/plant (a third of the highest yield) was from the low irrigation regime coupled with medium air temperature, a 150 kg/ha N, and loamy sand soils. The decrease in yield could mainly be due to retarded growth, as parameters such as plant height and the number of branches were relatively low under decreased air temperature, irrigation regimes, and the nitrogen application rate on loamy sand soil (Table 2). This emphasizes the importance of proper crop management practices to maximize yield. It is worth noting that sandy loam soil combined with low irrigation and a 200 kg ha^−1^ N application rate under high temperature was much better than loamy sand soil in enhancing the herbage yield because sandy loam soils have a high water holding capacity, are nutrient efficient, and the water does not dry out quickly. This study depicted that the maximum herbage yield was supported by sandy loam soil. Sandy loamy soil similarly resulted in significantly increased growth characters and biomass yield of rosemary plants [60]. The increase in the fresh herbage yield observed with nitrogen fertilization is in accordance with results reported in oregano [33] and marjoram [61]. As recorded for lemon balm [44], high air temperatures were shown to be optimal for higher productivity of marjoram plants. On average, a medium irrigation regime gave increased herbage yield. La Bella et al. [62] also observed the best performances with *Rosmarinus officinalis* when irrigation was more frequent. Although no clearly discernible trend in the interaction was observed in this study, the results indicated that different factors could interactively influence herbage yield.

#### 2.1.6. Leaf Area

There were highly significant differences in the leaf area as affected by an interaction between air temperature and irrigation regimes, nitrogen application rates, and soil composition type (Table 1). The highest average leaf area per plant per harvest (243.1 cm^2^) was obtained under low irrigation and high air temperature levels linked with a nitrogen application rate of 200 kg/ha N to sandy loam soils (Table 7). It is noteworthy that this is the same treatment combination that gave the highest herbage yield (Table 6), suggesting that factors causing an increase in the leaf area may also improve herbage yield. The increase in the leaf area when increasing the nitrogen application rate in some cases may be attributed to the weakness of this element and its role in increasing the division of meristematic cells, which is reflected positively on the increase in the size of the vegetative system, including the height of the plant. Ghani et al. [63] also observed that increasing nitrogen fertilizer increased the leaf area of *Sorghum bicolor*. L. In the present study, sandy loam soil enhanced the leaf area of marjoram plants. Growth enhancement was also recorded in sandy loam soil with *Hibiscus sabdariffa* [64].

#### 2.1.7. Correlation between Plant Growth and Physiological Traits

Significant positive and negative correlations were observed among plant growth parameters (plant height, number of branches, herbage yield, and leaf area) and physiological traits (chlorophyll content and leaf temperature) (Table 8). The plant height had a relatively weak but statistically significant correlation with the number of branches (r = 0.247, *p* ≤ 0.05) and leaf temperature (r = −0.337, *p* ≤ 0.01). Similarly, the number of branches was weakly but significantly correlated to chlorophyll content (r = 0.241, *p* ≤ 0.05) and leaf area (r = 0.236, *p* ≤ 0.05). Strong and significantly positive correlations were established between the herbage yield and leaf area (r = 0.806, *p* ≤ 0.001), followed by the leaf area and chlorophyll content (r = 0.572, *p* ≤ 0.001), while the number of branches had a strong but significantly negative correlation with leaf temperature (r = −0.528, *p* ≤ 0.001). Thus, increased leaf chlorophyll content and reduced leaf temperature per unit leaf area may be of advantage in the search for higher herbage yields. Such physiological traits can potentially be used for modeling marjoram crop productivity with a generic model such as the Crop Environment Resource Synthesis (CERES) model [65].

### 2.2. Essential Oil Yield and Composition

*Origanum syriacum* essential oil yield and composition is known to be affected by altitude, region, and time of harvest, as well as the part of the plant [21]. As part of the study, the effect of varying air temperature and irrigation regimes, nitrogen fertilizer application, and soil type on the essential oil yield and composition was evaluated. The highest marjoram essential oil yield (g per kg of herbage material), when analysed for four harvests across the entire experimental period, was recorded in harvest two (0.90 g), followed by harvest one (0.68 g), while the lowest yields were observed in harvests three (0.51 g) and four (0.36 g). In terms of essential oil percentage, this corresponds to 0.09, 0.06, 0.05, and 0.03% for harvests two, one, three, and four, respectively (Table 9). This observed variability in the yield production of the essential oil extracted from *O. marjorana* cultivated in different conditions could be related to the interaction between harvesting period, edaphic factors, nutrient availability, irrigation, and air temperature management. 

Overall, the best performing treatment combinations that resulted in the highest oil yield were a high temperature regime, combined with low levels of irrigation and high levels of fertilization, on loamy sand soils. The essential oil composition varied considerably across the four experimental harvests. The three main constituents in harvest one were terpinene-4-ol (26.68%), γ-terpinene (9.90%), and β-caryophyllene (5.05%); in harvest two they were terpinene-4-ol (22.63%), (Z)-β-terpineol (16.60%), and linalyl acetate (8.66%); in harvest three they were terpinene-4-ol (26.21%), (Z)-β-terpineol (13.72%), and β-caryophyllene (11.04%); while in the fourth harvest, they were terpinene-4-ol (36.72%), γ-terpinene (10.28%), and (Z)-β-terpineol (10.85%). Generally, the most abundant constituents in marjoram essential oil were terpinene-4-ol [20,66] and (Z)-β-terpineol. However, terpinene-4-ol content was above the International Organization for Standardization (ISO) limits of acceptability, while no reference limits are available for (Z)-β-terpineol. The third and fourth most abundant constituents were γ-terpinene, which had no reference limitation available, and β-caryophyllene, but its quantity was outside the range of ISO standard of acceptability. Milenković et al. [67] reported a 31.15% terpinen-4-ol chemotype, present in North Africa. Kimera et al. [68] similarly observed 27.11–32.38% terpinen-4-ol within marjoram grown in Cairo, after soil fertilization with Nile tilapia aquaculture effluent water. Terpinen-4-ol was a significant component in an ocular acaricide formulation [69].

The results highlight the importance of climatic conditions in the cultivation of aromatic plants, since these factors influence the presence of certain enzymes responsible for the increase or decrease of certain components. In addition, the interaction of medium levels of irrigation and air temperature with a nitrogen fertilizer application rate of 150 kg/ha N to sandy clay loam soil recorded the highest terpinene-4-ol content (36.72%) in the fourth harvest. According to our results, it seems that chemical composition including the major constituents across the different harvests varied with the physiological stage of the plant. 

## 3. Materials and Methods

### 3.1. Experimental Site and Growth Conditions

A pot trial was conducted inside glasshouse compartments at the Agricultural Research Council-Vegetable, Industrial and Medicinal Plants (ARC-VIMP) research station in Roodeplaat, Pretoria, South Africa (latitude 25°59′ S, longitude 28°35′ E, and altitude of 1244 m), from September 2020 to May 2022. The compartments were equipped with a fan and a heater to maintain the pre-defined temperature levels in the glasshouses. The daily average air temperature varied from 16.7 to 36.6 °C in the high regime, 12.6 to 33.1 °C in the medium regime, and 11.0 to 26.7 °C in the low regime (Figure S1). This translated to daily air temperature averages of 28.9, 24.4, and 20.8 °C for the high, medium, and low temperature regimes, respectively. The daily relative humidity averages were 86.8, 81.4, and 77.6% in the high, medium, and low temperature regime glasshouses, respectively.

### 3.2. Plant Material

Marjoram (*Origanum marjorana*) plantlets were regenerated from stem cuttings of marjoram plants (accession number = ARC-EO 21) maintained at the essential oil crop genebank of the Agricultural Research Council in South Africa. Stem cuttings were grown on a mist bed (1:1 silica sand to peat volume ratio as the growing medium) in a tunnel at the ARC-VIMP research station. Thirty-five days old plantlets were transplanted on 4 September 2020 into 5-L plastic pots (25 cm in diameter × 21.5 cm in height) filled with hot air-dried sterilized soil.

### 3.3. Treatments and Experimental Design

The plantlets were grown under different regimes of air temperature averaging 20.9 (low), 24.4 (medium), and 28.9 °C (high), irrigation (up to 20, 40, and 60% soil water depletion from field capacity), soil fertilization (100, 150, and 200 kg N ha^−1^), and soil type (sandy loam, sandy clay loam, and loamy sand). The treatments were laid out with a factorial arrangement in a randomized complete block design with three replicates.

### 3.4. Soil Sampling, Analysis and Fertilizer Application

Three open-field sites were assigned at the ARC-VIMP research station for collection of the experimental soils. Soil samples were randomly taken from depths of 0–20 and 20–40 cm to assess chemical and physical properties (Table 10 and Table 11). The nitrogen (N) treatment levels were applied using the inorganic fertilizer limestone ammonium nitrate (28% N). The three nitrogen fertilizer rates were applied per plant (6, 9 and 13 g/plant N). Weed control was performed manually when necessary.

### 3.5. Soil Water Content Monitoring and Irrigation Scheduling

The frequency and amount of irrigation was determined based on soil water content monitoring using 10HS automatic capacitative water sensors, which were inserted at a depth of 15 cm within the root zone of the crop and connected to an Em50 data logger (Campbell Scientific Inc., Logan, UT, USA). Three irrigation frequency treatments were imposed consisting of irrigation every day, once at 09h00 (low regime), twice at 09h00 and 12h00 (medium regime), and three times at 09h00, 12h00, and 15h00 (high regime) for 4 to 6 min each time. Water meters (NetaFim, Cape Town, South Africa) were installed in the main line of each irrigation regime to monitor irrigation volumes. Water holding capacities of the growing media were 36.5, 34.8, and 34.1% at pot capacity for sandy loam, sandy clay loam, and loamy sand soils, respectively. The volume of water that was required to refill the pots to field capacity for each irrigation event (water volume depleted through evapotranspiration) was determined by supplying an excess volume and subtracting the volume of drained water from the applied volume. Before applying irrigation treatments, the plants were allowed to grow for a month until they had completely established. A computer-controlled drip irrigation system (NetaFim, Cape Town, South Africa) with water discharge rate of 32 mL per minute and a pressure range of 150–200 kPa was used and administered through one emitter per plant. During the experimental period (September 2020 to May 2022), treatments with the highest irrigation level received a cumulative irrigation amount of 244 L/plant, while the medium and low irrigation treatments received 163 and 82 L/plant, respectively (Figure S2). This resulted in the highest volumetric soil water content under the high irrigation treatment (0.275–0.396 m^3^/m^3^), followed by the medium irrigation treatment (0.168–0.347 m^3^/m^3^), while the low irrigation treatment had the least values (0.031–0.307 m^3^/m^3^) as illustrated in Figure S3. The average volumetric soil water content in the high, medium, and low irrigation regimes were 0.373, 0.315, and 0.149 m^3^/m^3^, respectively.

### 3.6. Plant Growth and Physiological Measurements

Plant growth and physiological data collected in this study included plant height, number of branches per plant, chlorophyll content, leaf temperature, leaf area and herbage yield. Plant height was measured from the soil surface to the tip of the tallest flowering stem using a tape measure. The numbers of branches were counted manually per plant. The leaf chlorophyll content was measured on a fully matured leaf, with a chlorophyll content meter (SPAD 502 plus, Konica Minolta, Japan). The leaf temperature was also measured on a fully matured leaf, using an infrared thermometer (Agriexpo, Anaheim, CA, USA). The herbage weight was determined by harvesting three replications of the same treatment by weighing the fresh plant material with PGL 2002 Adam scale (Long Branch, NJ, USA). Leaf area was measured using a portable laser leaf area meter (CI-202, ICT International^TM^, Armidale, NSW, Australia). These growth parameters were recorded at every harvest (25 March 2021, 22 July 2021, 13 December 2021, and 12 May 2022) for the duration of the experiment. Each treatment was replicated three times and all the three replications were sampled to enable statistical analysis. The plants were harvested at 10 cm above the soil in order to allow for the recovery of biomass production.

### 3.7. Essential Oil Extraction and Yield Evaluation

The essential oil was extracted from the leaves using a custom-built steam distillation unit. About 500 g of fresh plant material (leaves and flowers) was distilled for oil at a temperature of ±90 °C for 90–120 min until no more oil was recovered. The essential oil yield was calculated in terms of percentage, by measuring the volume of oil extracted per weight of fresh plant material. The essential oil samples were stored in vials sealed with Teflon-faced septa and kept at 4 °C in the dark until GC–MS analysis.

### 3.8. GC–MS Analysis

Determination of essential oil composition and identification of compounds was performed by gas chromatography–mass spectrometry (GC–MS). The GC–MS analysis was carried out using an Agilent Technologies 7890B (Agilent Technologies, Santa Clara, CA, USA) gas chromatograph coupled to an Agilent Technologies MS 5977A capillary column (29.6 m × 0.25 mm, 0.25 um film thickness). The following temperature program was used: initial temperature at 60 °C, heating by a rate of 3 °C/min up to 250 °C, followed by a heating rate of 20 °C/min up to 280 °C final temperature, which was maintained for 0.17 min, while injector and detector temperatures were set at 250 °C. Helium (constant flow rate of 0.66208 µL/min) was used as the carrier gas. Injection volume was 1 µL in splitless mode. Prior to injection, essential oil sample was diluted (1 µL essential oil/999 µL Hexane (dilution 1:1000)). Acquisition mass range was 40–400 *m/z*, and ionization voltage was 70 eV. Marjoram essential oil samples were analysed for each harvest (harvests 1, 2, 3, and 4) during the entire experimental period, from September 2020 to May 2022.

### 3.9. Statistical Analysis

Statistical analysis of variance (ANOVA) was performed on the observations of marjoram plants for the three temperatures combined [70] after testing the homogeneity of the experimental error variances using Bartlett’s test [71]. The residuals were examined for deviations from normality, and outliers causing skewness were removed. Fisher’s least significant difference (LSD) was calculated at the 5% level to compare means for significant effects [72]. Analysis was performed using Genstat for Windows 18^th^ Edition (VSN International, 2015). Where interaction effects were significant, the highest combinations of factors having significant interaction effects were used to explain the results. On the other hand, where there were no significant interaction effects, the individual effects were used to explain the findings. Correlation coefficient (r) values between all plant growth and physiological traits investigated were determined by the Pearson correlation matrix method using XLSTAT software (ver. 17.04.36025 Add-in-soft, New York, NY, USA).

## 4. Conclusions

This study demonstrated differences in the productivity and chemical composition of marjoram plants in response to air temperature and irrigation regimes, nitrogen fertilizer application rate and soil type, and their interactions during crop growth and development. Research findings showed the optimum productivity of marjoram under conditions of high air temperatures, low irrigation, and high to medium nitrogen fertilizer applied to sandy loam soils, with increased oil yield under loamy sand soils. A promising performance of marjoram cultivation under high regimes of air temperature (16.7 to 36.6 °C), nitrogen fertilization (200 kg ha^−1^ N), and low irrigation (128–192 mL per plant per day) on loamy sand soils was demonstrated for improved crop productivity and essential oil quality. For better inferences, however, it is suggested that these findings be validated under field environmental conditions. Future research may also incorporate the micro details (perhaps using scanning electron microscope) of trichome types, their density, and their distribution in various plant treatment samples.

## Figures and Tables

**Table 1:** Analysis of variance for the effect of air temperature and irrigation regimes, nitrogen application rate, and soil type on plant height, number of branches, leaf chlorophyll content, leaf temperature, herbage yield, and leaf area of marjoram plants.

Source of Variation	df	Plant Height		Number of Branches		Chlorophyll Content		Leaf Temperature		Herbage Yield		Leaf Area	
		MS	*p*-Value	MS	*p*-Value	MS	*p*-Value	MS	*p*-Value	MS	*p*-Value	MS	*p*-Value
Temp	2	143.6ns	0.182	30.8ns	0.060	41.9 **	0.004	0.1ns	0.800	843.7 ***	<0.001	103.4 ***	<0.001
Irri	2	351.0 **	0.007	204.1 ***	<0.001	193.1 ***	<0.001	40.4 ***	<0.001	22596.5 ***	<0.001	2783.8 ***	<0.001
Soil	2	714.5 ***	<0.001	3.0ns	0.279	2.6ns	0.498	1.6ns	0.236	645.3 ***	<0.001	79.7 ***	<0.001
Fert	2	4.6ns	0.877	16.4 ***	0.001	122.4 ***	<0.001	9.9 ***	<0.001	27862.7 ***	<0.001	3434.9 ***	<0.001
Irri × Temp	4	114.1ns	0.094	11.2ns	0.175	37.1 **	0.002	0.7ns	0.528	1754.7 ***	<0.001	216.5 ***	<0.001
Irri × Soil	4	46.4ns	0.264	3.2ns	0.240	12.1 *	0.013	0.4ns	0.803	105.9ns	0.101	13.1ns	0.099
Irri × Fert	4	233.7 ***	<0.001	6.1 *	0.037	144.7 ***	<0.001	0.3ns	0.856	5864.0 ***	<0.001	723.6 ***	<0.001
Temp × Soil	4	98.2 *	0.028	3.0ns	0.270	42.6 ***	<.001	0.5ns	0.733	645.8 ***	<0.001	79.6 ***	<0.001
Temp × Fert	4	83.3ns	0.055	6.3 *	0.034	22.1 ***	<.001	2.1ns	0.118	406.8 ***	<0.001	50.2 ***	<0.001
Soil × Fert	4	11.4ns	0.860	0.2ns	0.975	7.2ns	0.105	0.5ns	0.774	46.9ns	0.479	5.7ns	0.486
Irri × Temp × Soil	8	76.7 *	0.032	5.8 *	0.014	5.9ns	0.132	1.1ns	0.455	91.9ns	0.099	11.2ns	0.102
Irri × Temp × Fert	8	185.4 ***	<0.001	1.6ns	0.691	11.0 **	0.004	2.2ns	0.057	536.8 ***	<0.001	66.3 ***	<0.001
Irri × Soil × Fert	8	52.6ns	0.163	1.3ns	0.794	17.1 ***	<0.001	1.6ns	0.184	471.4 ***	<0.001	58.0 ***	<0.001
Temp × Soil × Fert	8	130.7 ***	<0.001	2.0ns	0.532	3.9ns	0.386	1.0ns	0.485	351.0 ***	<0.001	43.3 ***	<0.001
Irri × Temp × Soil × Fert	16	56.0ns	0.077	0.8ns	0.986	15.6 ***	<0.001	0.7ns	0.795	516.8 ***	<0.001	63.8 ***	<0.001
Residual	144	35.1		2.3		3.71		1.1		53.5		6.6	
Total	242												

*p*-value = probability level based on Fisher’s least significant difference test, MS = mean square, df = degree of freedom, *** = p ≤ 0.001, ** = *p* ≤ 0.01, * = *p* ≤ 0.05, ns = not significant, Temp = temperature, Irri = irrigation, Soil = soil type, Fert = fertilizer.

**Table 2:** Plant height (cm) as influenced by interaction effects of (i) nitrogen fertilizer application rate, air temperature, and soil type, (ii) nitrogen fertilizer application rate, air temperature, and irrigation regime, and (iii) irrigation regime, temperature, and soil type.

**Research Treatments**		**Temperature Regime**	**Fertilization Rate (kg ha^−1^)**		
			**100**	**150**	**200**
Soil type	Sandy clay loam	High	40.66 b–f	41.21 b-e	33.09 hi
		Medium	39.83 b–g	39.66 b–f	44.64 ab
		Low	37.40 d–h	39.04 b–g	42.59 a–d
	Loamy sand	High	35.96 e–i	36.27 e–i	36.40 e–i
		Medium	36.11 e–i	36.09 e–i	34.49 g–i
		Low	32.87 hi	34.37 g–i	30.64 i
	Sandy loam	High	47.17 a	38.23 c–h	43.17 a–d
		Medium	38.89 c–g	43.41 a–c	36.53 e–h
		Low	34.98 f–i	37.97c–h	40.40 b–f
Irrigation regime	Low	High	42.16 a–d	36.83 c–i	30.80 jk
		Medium	38.43 b–h	34.59 g–k	42.28 a–c
		Low	31.37 i–k	30.87 jk	36.20 d–j
	Medium	High	39.69 b-g	39.86 b–g	41.74 a–f
		Medium	29.61 k	41.02 a–f	39.73 b–g
		Low	36.27 d–j	41.83 a–e	38.51 b–h
	High	High	41.93 a–d	39.02 b–h	40.11 b–g
		Medium	46.79 a	43.54 ab	33.66 h–k
		Low	37.61 b–h	38.68 b–h	38.92 b–h
**Research Treatments**		**Temperature Regime**	**Irrigation Regime**		
			**Low**	**Medium**	**High**
Soil type	Sandy clay loam	High	36.88 d–g	38.03 c–g	40.04 b–f
		Medium	35.80 e–h	41.72 a–d	46.61a
		Low	36.37 d–h	41.79 a–d	40.88 a–e
	Loamy sand	High	33.40 g–i	37.46 c–g	37.77 c–g
		Medium	40.22 b–f	30.53 hi	35.93 d–h
		Low	28.59 i	34.74 f–h	34.54 f–h
	Sandy loam	High	39.51 c–f	45.80 ab	43.26 a–c
		Medium	39.28 c–g	38.11 c–g	41.44 a–e
		Low	33.48 g–i	40.08 b–f	39.79 c–f

Mean values per combination of factors [(i) fertilization rate, temperature, and soil type (data presented in black font); (ii) fertilization rate, temperature, and irrigation regime (data presented in green font); (iii) irrigation regime, temperature, and soil type (data presented in blue font)] having a common letter within an alphabetical order range are not significantly different based on Fisher’s least significant difference test (*p* > 0.05).

**Table 3:** Interactive effect of soil type, air temperature and irrigation regimes on number of branches.

Soil Type	Temperature Regime	Irrigation Regime		
		Low	Medium	High
Sandy clay loam	High	5.02 i	5.34 hi	8.77 a–c
	Medium	4.58 i	4.34 i	7.83 b–d
	Low	4.58 i	6.94 d–h	7.32 c–f
Loamy sand	High	4.97 i	5.57 f–i	9.20 ab
	Medium	4.93 i	4.64 i	6.10 e–i
	Low	5.72 e–i	5.87 e–i	6.83 d–h
Sandy loam	High	4.97 i	7.21 c–g	9.77 a
	Medium	4.91 i	4.71 i	7.50 b–e
	Low	5.27 hi	4.58 i	8.13 a–d

Mean values having a common letter within an alphabetical order range are not significantly different based on Fisher’s least significant difference test (*p* > 0.05).

**Table 4:** Interactive effect of air temperature and irrigation regimes, nitrogen fertilizer application, and soil type on chlorophyll content (SPAD).

Irrigation Regime	Fertilization (kg ha^−1^)	Soil Type	Temperature Regime		
			Low	Medium	High
High	100	Loamy sand	34.27 a–e	31.10 f–n	31.17 e–n
		Sandy clay loam	29.80 j–r	29.83 j–r	32.77 a–k
		Sandy loam	34.97 a–c	29.40 l–s	30.33 h–p
	150	Loamy sand	32.40 a–l	32.77 a–k	33.17 a–i
		Sandy clay loam	30.63 f–p	27.13 q–x	32.63 a–k
		Sandy loam	33.43 a–h	31.93 c–m	30.97 f–o
	200	Loamy sand	30.07 i–r	33.50 a–g	29.93 j–r
		Sandy clay loam	29.70 k–r	30.13 i–r	34.40 a–d
		Sandy loam	30.13 i–q	28.43 n–t	27.03 q–y
Medium	100	Loamy sand	30.07 i–r	33.50 a–g	29.93 j–r
		Sandy clay loam	29.70 k–r	30.13 i–r	34.40 a–d
		Sandy loam	30.13 i–q	28.43 n–t	25.50 t–y
	150	Loamy sand	27.57 p–w	27.17 q–x	30.47 g–p
		Sandy clay loam	28.40 n–u	31.00 f–o	27.90 o–v
		Sandy loam	31.00 f–o	23.93 y	29.70 j–r
	200	Loamy sand	31.50 d–n	31.77 d–m	27.53 p–w
		Sandy clay loam	31.10 f–n	29.97 j–r	32.63 a–k
		Sandy loam	31.50 d–n	32.20 a–l	34.40 a–d
Low	100	Loamy sand	24.13 xy	24.53 w–y	24.57 w–y
		Sandy clay loam	24.30 xy	25.17 v–y	24.57 w–y
		Sandy loam	24.70 w–y	24.40 xy	24.47 w–y
	150	Loamy sand	25.27 u–y	28.93 m–s	29.43 l–s
		Sandy clay loam	24.67 w–y	27.00 r–y	35.27 a
		Sandy loam	24.63 w–y	26.43 s–y	32.03 b–m
	200	Loamy sand	27.20 q–x	31.40 d–n	32.83 a–j
		Sandy clay loam	29.77 j–r	31.43 d–n	33.63 a–f
		Sandy loam	32.33 a–l	31.33 d–n	35.13 ab

Mean values having a common letter within an alphabetical order range are not significantly different based on Fisher’s least significant difference test (*p* > 0.05).

**Table 5:** The effect of nitrogen fertilizer application rate and irrigation regime on leaf temperature.

**Parameter**	**Nitrogen Fertilization Rate (kg ha^−1^)**		
	100	150	200
Leaf temperature (°C)	18.06 a	17.87 a	17.38 b
	**Irrigation Regime**		
	Low	Medium	High
Leaf temperature (°C)	18.25 a	18.09 a	16.96 b

Mean values per row having a common letter are not significantly different based on Fisher’s least significant difference test (*p* > 0.05).

**Table 6:** Interactive effect of air temperature and irrigation regimes, nitrogen fertilizer application, and soil type on herbage yield (g/plant).

Irrigation Regime	Fertilization (kg ha^−1^)	Soil Type	Temperature Regime		
			Low	Medium	High
	100	Loamy sand	59.3 JK	87.0 x–A	90.3 w–z
		Sandy clay loam	92.7 v–y	85.3 y–C	82.0 y–E
		Sandy loam	85.7 y–C	84.3 y–C	97.7 u–x
High	150	Loamy sand	84.7 y–C	74.3 C–G	91.3 w–z
		Sandy clay loam	62.7 H–K	72.0 E–H	89.0 w–A
		Sandy loam	70.0 F–I	99.3 t–w	106.3 r–u
	200	Loamy sand	126.7 g–l	136.3 c–h	124.3 i–n
		Sandy clay loam	138.0 b–g	120.7 j–p	137.3 b–g
		Sandy loam	116.7 l–r	124.7 h–n	144.7 b–d
	100	Loamy sand	146.3 a–c	111.3 o–s	114.3 m–s
		Sandy clay loam	148.3 ab	104.0 s–v	105.0 s–u
		Sandy loam	108.3 q–u	104.3 s–u	132.0 e–j
Medium	150	Loamy sand	125.3 h–m	127.0 g–l	118.0 k–q
		Sandy clay loam	117.3 l–r	126.7 g–l	117.0 l–r
		Sandy loam	133.7 d–i	128.0 f–l	139.0 b–f
	200	Loamy sand	141.3 b–e	126.7 g–l	113.3 n–s
		Sandy clay loam	121.7 j–o	131.0 e–j	118.0 k–q
		Sandy loam	144.7 b–d	126.7 g–l	109.3 p–t
	100	Loamy sand	71.0 E–I	75.3 B–G	90.7 w–z
		Sandy clay loam	68.7 G–J	77.7 A–G	72.3 D–H
		Sandy loam	85.3 y–C	80.7 z–F	86.0 y–B
Low	150	Loamy sand	71.7 E–I	53.3 K	84.0 y–D
		Sandy clay loam	90.0 w–z	60.3 I–K	86.7 x–B
		Sandy loam	56.3 K	71.7 E–I	84.3 y–C
	200	Loamy sand	108.0 q–u	131.7 e–j	112.3 o–s
		Sandy clay loam	109.0 q–u	118.3 k–q	129.0 f–k
		Sandy loam	111.7 o–s	131.0 e–j	157.3 a

For mean value comparisons, mean values are followed by alphabetical letters (starting with lower case letters a to z, followed by upper case letters A to Z). Mean values that have a common lower case or upper case letter within an alphabetical order range are not significantly different based on Fisher’s least significant difference test (*p* > 0.05). Lower and upper case letters serve to differentiate treatment means.

**Table 7:** Interactive effect of air temperature and irrigation regimes, nitrogen fertilizer application, and soil type on leaf area (cm^2^/plant).

Irrigation Regime	Fertilization (kg ha^−1^)	Soil Type	Temperature Regime		
			Low	Medium	High
	100	Loamy sand	208.6 JK	218.3 x–A	219.5 w–z
		Sandy clay loam	220.3 v–y	217.7 y–C	216.6 y–E
		Sandy loam	217.9 y–C	217.4 y–C	222.1 u–x
High	150	Loamy sand	217.5 y–C	213.9 C–G	219.8 w–z
		Sandy clay loam	209.8 H–K	213.0 E–H	219.0 w–A
		Sandy loam	212.3 F–J	222.6 t–w	225.1 r–u
	200	Loamy sand	232.2 g–l	235.6 c–h	231.4 i–n
		Sandy clay loam	236.2 b–g	230.2 j–p	236.0 b–g
		Sandy loam	228.7 l–r	231.6 h–n	238.6 b–d
	100	Loamy sand	239.1 a–c	226.9 o–s	227.9 m–s
		Sandy clay loam	239.9 ab	224.3 s–v	224.6 s–u
		Sandy loam	225.8 q–u	224.4 s–u	234.1 e–j
Medium	150	Loamy sand	231.8 h–m	232.4 g–l	229.2 k–q
		Sandy clay loam	229.0 l–r	232.3 g–l	228.8 l–r
		Sandy loam	234.7 d–i	232.7 f–l	236.6 b–f
	200	Loamy sand	237.4 b–e	232.3 g–l	227.6 n–s
		Sandy clay loam	230.5 j–o	233.8 e–j	229.2 k–q
		Sandy loam	238.5 b–d	232.3 g–l	226.1 p–t
	100	Loamy sand	212.7 E–I	214.2 B–G	219.6 w–z
		Sandy clay loam	211.9 G–J	215.1 A–G	213.2 D–H
		Sandy loam	217.7 y–C	216.1 z–F	218.0 y–B
Low	150	Loamy sand	212.9 E–I	206.5 K	217.3 y–D
		Sandy clay loam	219.4 w–z	208.9 I–K	218.2 x–B
		Sandy loam	207.6 K	212.9 E–I	217.4 y–C
	200	Loamy sand	225.7 q–u	234.0 e–j	227.2 o–s
		Sandy clay loam	226.0 q–u	229.3 k–q	233.1 f–k
		Sandy loam	227.0 o–s	233.8 e–j	243.1 a

For mean value comparisons, mean values are followed by alphabetical letters (starting with lower case letters a to z, followed by upper case letters A to Z). Mean values that have a common lower case or upper case letter within an alphabetical order range are not significantly different based on Fisher’s least significant difference test (*p* > 0.05). Upper and lower case letters serve to differentiate treatment means.

**Table 8:** Pearson’s correlation coefficient (r) between plant growth and physiological traits.

Trait	Plant Height	Number of Branches	Chlorophyll Content	Leaf Temperature	Herbage Yield
Number of branches	0.247 *				
Chlorophyll content	0.006ns	0.241 *			
Leaf temperature	−0.337 **	−0.528 ***	−0.339 **		
Herbage yield	0.102ns	0.075ns	0.303 **	−0.033ns	
Leaf area	0.121ns	0.236 *	0.572 ***	−0.274 *	0.806 ***

*** = p ≤ 0.001, ** = *p* ≤ 0.01, * = *p* ≤ 0.05, ns = not significant.

**Table 9:** Summary of oil yield, percentage, and composition of marjoram plants under varying air temperature and irrigation regimes, nitrogen application rate, and soil type.

Harvest Period	Best Performing Treatment under Varying Factors ^1^	Oil Yield (g/kg Herbage Yield)	Oil Percentage (%)	Oil Composition (% of Three Main Constituents)	ISO Standards (%) (ISO 4728:2003)
Harvest 1	High-Loamy sand-Low-High	0.68	0.06	Terpinene-4-ol (26.68)	0.2–1.2
				γ-Terpinene (9.90)	-
				β-Caryophyllene (5.05)	0.5–1.5
Harvest 2	High-Loamy sand-Low-High	0.90	0.09	Terpinene-4-ol (22.63)	0.2–1.2
				(Z)-β-Terpineol (16.60)	-
				Linalyl acetate (8.66)	0.2–4.0
Harvest 3	High-Loamy sand-Low-High	0.51	0.05	Terpinene-4-ol (26.21)	0.2–1.2
				(Z)-β-Terpineol (13.72)	-
				β-Caryophyllene (11.04)	0.5–1.5
Harvest 4	Medium-Sandy clay loam-Medium-Medium	0.36	0.03	Terpinene-4-ol (36.72)	0.2–1.2
				γ-Terpinene (10.28)	-
				(Z)-β-Terpineol (10.85)	-

^1^ Best performing treatments under a combination of air temperature, soil type, irrigation, and fertilization. - = No references available in terms of the ISO standards.

**Table 10:** Chemical properties of the experimental soils.

Chemical Elements	Units	Sandy Clay Loam		Loamy Sand		Sandy Loam	
		Soil Depth (cm)					
		0–20	20–40	0–20	20–40	0–20	20–40
pH (H_2_O)	-	7.12	7.14	6.57	6.21	5.83	5.80
EC	(ms m^−1^)	4.10	4.10	2.60	2.50	2.90	2.80
Boron (B)	(mg kg^−1^)	0.04	0.03	0.01	0.01	0.02	0.03
Iron (Fe)	(mg kg^−1^)	15.00	16.40	30.60	35.20	16.20	22.20
Manganese (Mn)	(mg kg^−1^)	188.00	199.00	27.70	31.80	43.90	39.20
Copper (Cu)	(mg kg^−1^)	2.35	2.41	0.85	0.74	1.01	1.08
Zinc (Zn)	(mg kg^−1^)	3.84	4.49	2.93	3.14	2.65	2.03
Chlorine (Cl)	(mg kg^−1^)	2.17	2.18	1.58	0.42	1.15	1.66
Nitrate (NO_3_)	(mg kg^−1^)	1.87	1.84	1.65	0.37	1.78	1.77
Nitrogen dioxide (NO_2_)	(mg kg^−1^)	0.01	0.01	0.02	0.00	0.02	0.02
Phosphate ion (PO_4_)	(mg kg^−1^)	1.59	1.55	0.84	0.33	0.55	0.38

**Table 11:** Physical properties of the experimental soils.

Texture	Soil Depth (cm)	Particle Size			Bulk Density (g cm^−3^)
		Sand (%)	Silt (%)	Clay (%)	
Sandy clay loam	0–20	74.0	4.0	22.0	1.20
Sandy clay loam	20–40	72.0	6.0	22.0	1.16
Loamy sand	0–20	86.0	4.0	10.0	1.30
Loamy sand	20–40	88.0	4.0	8.0	1.26
Sandy loam	0–20	78.0	6.0	16.0	1.26
Sandy loam	20–40	78.0	4.0	18.0	1.30

## Data Availability

Not applicable.

## Supplementary Materials

The following supporting information can be downloaded at: https://www.mdpi.com/article/10.3390/plants12010173/s1, Figure S1: Daily average air temperature and relative humidity in the different glasshouse compartments, namely (a) high, (b) medium, and (c) low regimes during the entire plant growing season; Figure S2: Cumulative irrigation applied per marjoram plant in glasshouse compartments from September 2020 to May 2022; Figure S3: Changes in soil water content (SWC) within the crop root zone for the low, medium and high irrigation regimes.
