# Supplementary material for: Biomass, Essential Oil Yield, and Composition of Marjoram as Influenced by Interactions of Different Agronomic Practices under Controlled Conditions
Source: Plants (Basel). 2022 Dec 30;12(1):173. doi: 10.3390/plants12010173 (PMC9824495; doi:10.3390/plants12010173)
Supplement: Supplementary file 1 [file plants-12-00173-s001.zip › plants-2043778-supplementary.pdf]

plants

MDPI

Article

Biomass, Essential Oil Yield, and Composition of Marjoram as Influenced by Interactions of Different Agronomic Practices under Controlled Conditions

Mantwampe Johleen Malaka [1,2], Nadia Alcina Araya [1], Puffy Soundy [2], Christian Phillipus du Plooy [1], Hintsa Tesfamicael Araya [1], Willem Sternburg Jansen Van Rensburg [1], Eric Watkinson [3], Ellis Levember [4], Ebrahim Wadiwala [5] and Stephen Oluwaseun Amoo [1,6,7,*]

1 Agricultural Research Council-Vegetable, Industrial and Medicinal Plants, Private Bag X293, Pretoria 0001, South Africa
2 Department of Crop Sciences, Tshwane University of Technology, Private Bag X680, Pretoria 0001, South Africa
3 Department of Science and Innovation, Private Bag X894, Pretoria 0001, South Africa
4 South African Essential Oils Business Incubator, 19 Mountain Street, Derdepoort 0186, Pretoria, South Africa
5 Council for Scientific and Industrial Research, P.O. Box 395, Meiring Naude Road, Brummeria, Pretoria 0001, South Africa
6 Department of Botany and Plant Biotechnology, University of Johannesburg, P.O. Box 524, Auckland Park, Johannesburg 2006, South Africa
7 Indigenous Knowledge Systems Centre, Faculty of Natural and Agricultural Sciences, North-West University, Private Bag X2046, Mmabatho 2790, South Africa
* Correspondence: amoos@arc.agric.za

Citation: Malaka, M.J.; Araya, N.A.; Soundy, P.; du Plooy, C.P.; Araya, H.T.; Jansen Van Rensburg, W.S.; Watkinson, E.; Levember, E.; Wadiwala, E.; Amoo, S.O. Biomass, Essential Oil Yield, and Composition of Marjoram as Influenced by Interactions of Different Agronomic Practices under Controlled Conditions. *Plants* **2023**, *12*, 173. https://doi.org/10.3390/plants12010173

Academic Editors: Mariateresa Cardarelli and Othmane Merah

Received: 3 November 2022
Revised: 25 December 2022
Accepted: 27 December 2022
Published: 30 December 2022

Copyright: © 2022 by the authors. Licensee MDPI, Basel, Switzerland. This article is an open access article distributed under the terms and conditions of the Creative Commons Attribution (CC BY) license (https://creativecommons.org/licenses/by/4.0/).

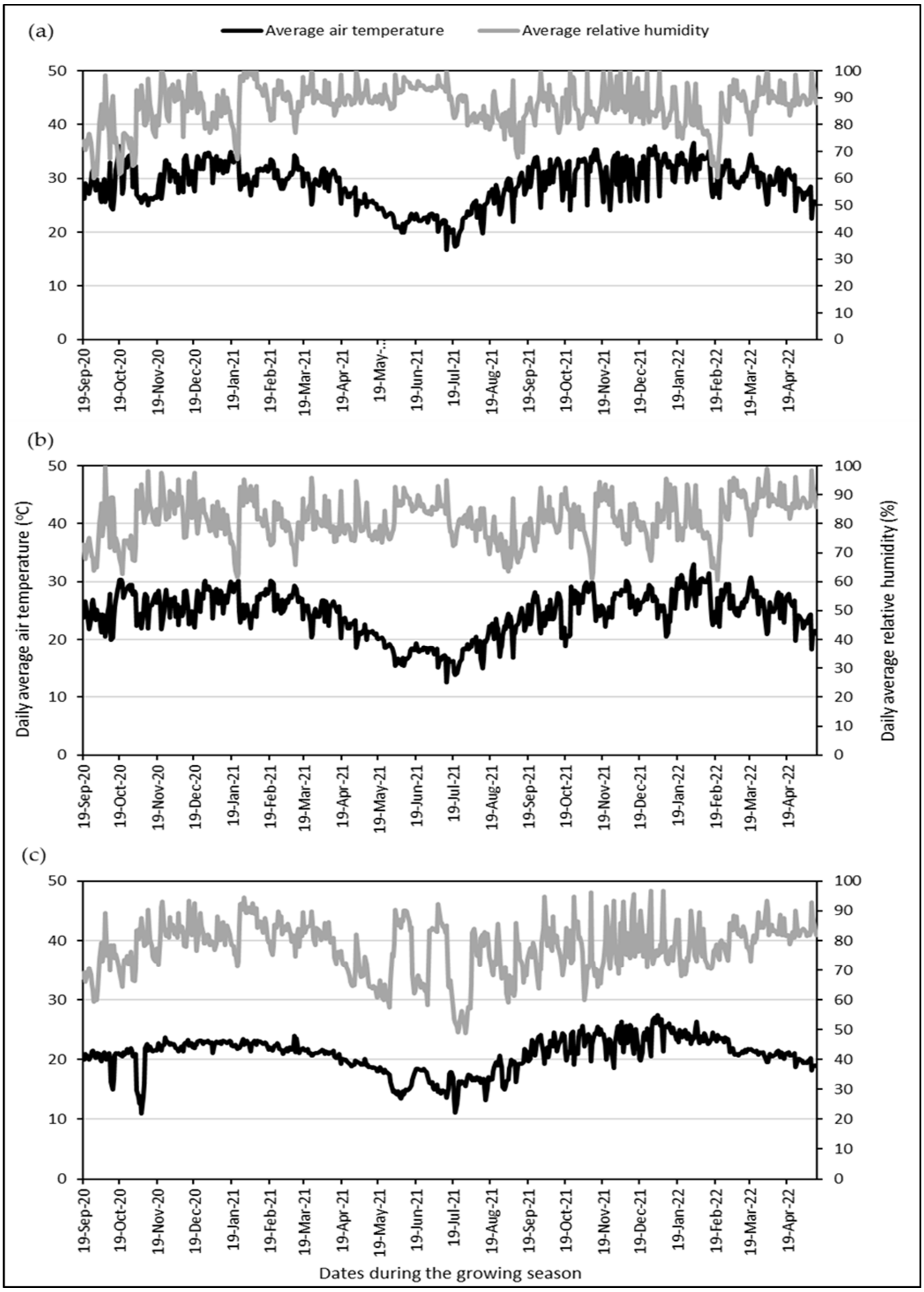

Figure S1. Daily average air temperature and relative humidity in the different glasshouse compartments, namely (a) high, (b) medium, and (c) low regimes during the entire plant growing season.

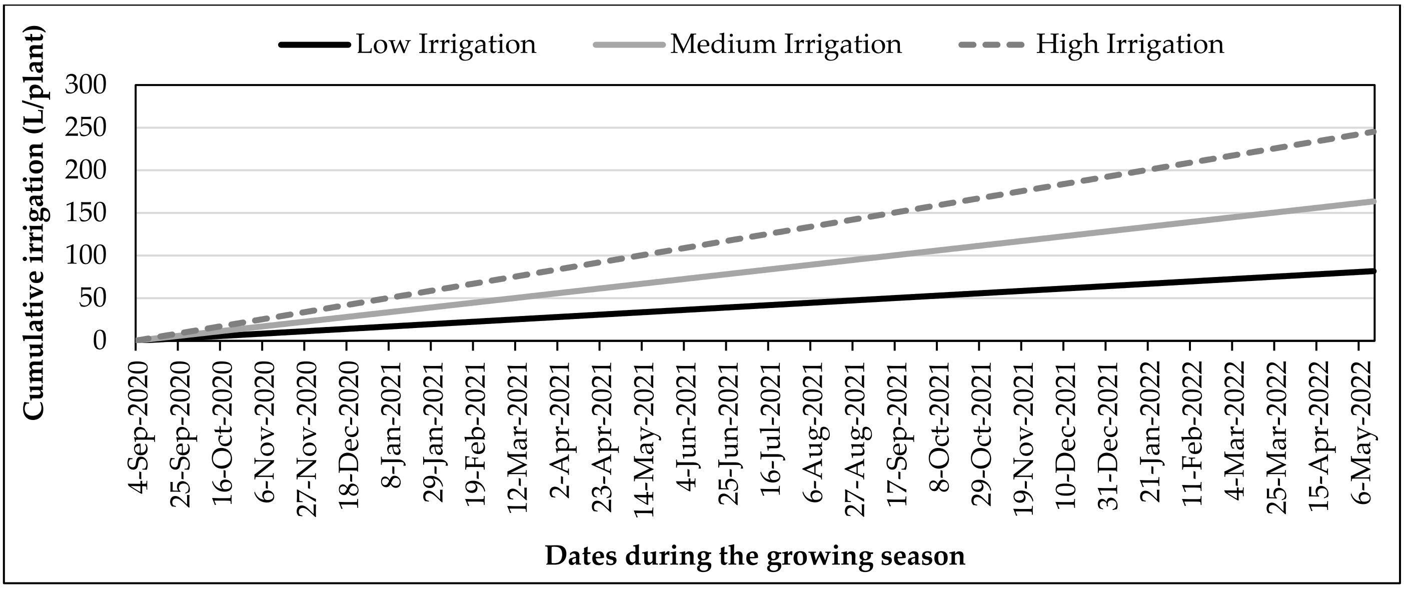

Figure S2. Cumulative irrigation applied per marjoram plant in glasshouse compartments from September 2020 to May 2022.

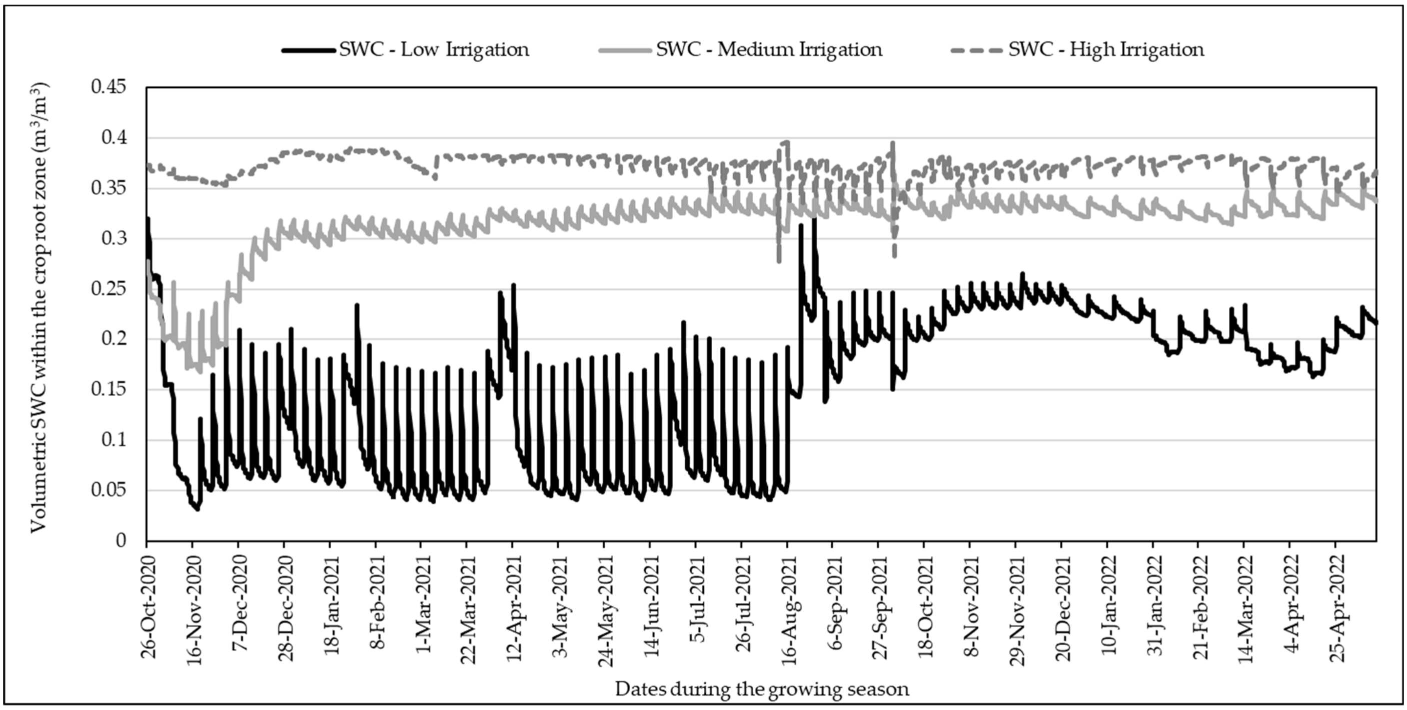

Figure S3. Changes in soil water content (SWC) within the crop root zone for the low, medium and high irrigation regimes.
